# Selections, frameshift mutations, and copy number variation detected on the *surf*_*4.1*_ gene in the western Kenyan *Plasmodium falciparum* population

**DOI:** 10.1186/s12936-017-1743-x

**Published:** 2017-03-02

**Authors:** Jesse N. Gitaka, Mika Takeda, Masatsugu Kimura, Zulkarnain Md Idris, Chim W. Chan, James Kongere, Kazuhide Yahata, Francis W. Muregi, Yoshio Ichinose, Akira Kaneko, Osamu Kaneko

**Affiliations:** 1 Graduate School of Biomedical Sciences, Nagasaki University, 1-12-4 Sakamoto, Nagasaki, 852-8523 Japan; 2 Department of Protozoology, Institute of Tropical Medicine (NEKKEN), Nagasaki University, 1-12-4 Sakamoto, Nagasaki, 852-8523 Japan; 3 Department of Clinical Medicine, Mount Kenya University, PO Box 342-01000, Thika, Kenya; 4 Radioisotope Centre, Graduate School of Medicine, Osaka City University, 1-4-3 Asahimachi, Abeno-ku, Osaka, 545-8585 Japan; 5 Island Malaria Group, Department of Microbiology, Tumor and Cell Biology (MTC), Karolinska Institutet, Nobels väg 16, SE 171 77 Stockholm, Sweden; 6 Department of Parasitology and Medical Entomology, Faculty of Medicine, Universiti Kebangsaan Malaysia Medical Centre, 56000 Kuala Lumpur, Malaysia; 7 Nairobi Research Station, Nagasaki University Institute of Tropical Medicine-Kenya Medical Research Institute (NUITM-KEMRI) Project, Institute of Tropical Medicine (NEKKEN), Nagasaki University, P. O. Box 19993-00202, Nairobi, Kenya; 8 Department of Parasitology and Research Center for Infectious Disease Sciences, Graduate School of Medicine, Osaka City University, 1-4-3 Asahimachi, Abeno-ku, Osaka, 545-8585 Japan; 9 Institute of Tropical Medicine (NEKKEN), Nagasaki University, Nagasaki, 1-12-4 Sakamoto, Nagasaki, 852-8523 Japan

**Keywords:** Copy number variation, Frameshift, Malaria, *Plasmodium falciparum*, Selection, SURFIN_4.1_

## Abstract

**Background:**

*Plasmodium falciparum* SURFIN_4.1_ is a putative ligand expressed on the merozoite and likely on the infected red blood cell, whose gene was suggested to be under directional selection in the eastern Kenyan population, but under balancing selection in the Thai population. To understand this difference, *surf*_*4.1*_ sequences of western Kenyan *P. falciparum* isolates were analysed. Frameshift mutations and copy number variation (CNV) were also examined for the parasites from western Kenya and Thailand.

**Results:**

Positively significant departures from neutral expectations were detected on the *surf*_*4.1*_ region encoding C-terminus of the variable region 2 (Var2) by 3 population-based tests in the western Kenyan population as similar in the Thai population, which was not covered by the previous analysis for eastern Kenyan population. Significant excess of non-synonymous substitutions per nonsynonymous site over synonymous substitutions per synonymous site was also detected in the Var2 region. Negatively significant departures from neutral expectations was detected on the region encoding Var1 C-terminus consistent to the previous observation in the eastern Kenyan population. Parasites possessing a frameshift mutation resulting a product without intracellular Trp-rich (WR) domains were 22/23 in western Kenya and 22/36 in Thailand. More than one copy of *surf*_*4.1*_ gene was detected in western Kenya (4/24), but no CNV was found in Thailand (0/36).

**Conclusions:**

The authors infer that the high polymorphism of SURFIN_4.1_ Var2 C-terminus in both Kenyan and Thai populations were shaped-up by diversifying selection and maintained by balancing selection. These phenomena were most likely driven by immunological pressure. Whereas the SURFIN_4.1_ Var1 C-terminus is suggested to be under directional selection consistent to the previous report for the eastern Kenyan population. Most western Kenyan isolates possess a frameshift mutation that would limit the expression of SURFIN_4.1_ on the merozoite, but only 60% of Thai isolates possess this frameshift, which would affect the level and type of the selection pressure against this protein as seen in the two extremities of Tajima’s *D* values for Var1 C-terminus between Kenyan and Thai populations. CNV observed in Kenyan isolates may be a consequence of this frameshift mutation to increase benefits on the merozoite surface.

## Background

Malaria poses a serious public health challenge causing estimated 438,000 deaths and 214 million clinical cases in 2015, especially in sub-Saharan Africa where the dominant species is *Plasmodium falciparum* [1]. In human hosts, *P. falciparum* multiplies in the red blood cell (RBC), where malaria pathogenicity is mediated by parasite-encoded proteins expressed on the RBC invasive merozoite stage parasite and parasite-infected RBC surface. Therefore, parasite proteins that interact with the host RBCs and endothelial cells are potential immune targets and may serve as vaccine candidates for intervention.

**Fig. 1:**
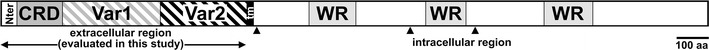
Schematic structure of SURFIN_4.1_. Extracellular region is divided into 4 parts; N-terminal (Nter), Cys-rich domain (CRD), and variable regions 1 and 2 (Var1 and Var2). *Scale bar* indicates 100 amino acids (aa). The length of the cytoplasmic region varies among parasite lines due to the frameshift mutations: 3D7 line SURFIN_4.1_ has a stop codon (*first arrowhead*, at nt 2498–2503) just after the transmembrane region (tm), another potential frameshift would generate a stop codon (*second arrowhead* at nt 3894–3903) before 2nd Trp-rich (WR) domain, FCR3 line has a stop codon (*third arrowhead* at nt 4529–4536) after 2nd WR domain [4], and IT line sequence has no frameshift mutations and contains three WR domains

A large type 1 transmembrane protein SURFIN encoded by a surface-associated interspersed gene (*surf* gene) family has a unique position in such proteins, because its extracellular Cys-rich domain (CRD) and intracellular Trp-rich (WR) domain have homology with a variety of adhesins expressed on the surface of the RBC infected by not only *P. falciparum* but also other *Plasmodium* species, for example, CRD with PIR proteins encoded by the *Plasmodium* interspersed repeats (*pir*) super multigene family in primate and rodent malaria parasites and WR with *P. falciparum* PfEMP1 and *Plasmodium knowlesi SICAvar* [2], suggesting SURFIN components were utilized to generate lineage-specific adhesins expressed on the iRBC surface through domain shuffling with other proteins. These shared structural components point to an evolutionary basal position and important roles of SURFIN in the parasite–host interaction. *P. falciparum* SURFIN_4.1_ is proposed to be involved in the reversible association with target RBCs when it is located on the merozoite surface [3]. Furthermore, exogenously expressed partial SURFIN_4.1_ in *P. falciparum* was observed to be transported to the iRBC [4], suggesting that this protein is potentially also expressed on the iRBC surface as its paralog SURFIN_4.2_ [2]. Interestingly, in *P. falciparum* 3D7 and MS822 lines, SURFIN_4.1_ is truncated just after the transmembrane region due to a premature stop codon caused by a frameshift mutation. Conversely, in another line FCR3, this frameshift mutation does not occur and SURFIN_4.1_ contains two WR domains in the intracellular region [4]. Moreover, the sequence of IT line *P. falciparum* in the PlasmoDB database (PFIT_0400900) does not possess these frameshifts and the protein product is expected to contain three WR domains similar to SURFIN_4.2_ (Fig. 1). Since the intracellular region containing WR domain has an important role for the iRBC surface expression in the case of SURFIN_4.2_ [5], SURFIN_4.1_ would be expressed on the iRBC surface only by *P. falciparum* isolates possessing WR domain. SURFIN_4.1_ variants without WR domain would be expressed only on the merozoite surface. The abundance of such type of SURFIN_4.1_ would affect the evolutionary process and diversity as the exposure times to the host immune system are very different (>24 h on the iRBC surface versus up to several minutes on the merozoite surface). Optimal specificity and strength of the putative SURFIN_4.1_-host receptor interactions, although not proved yet, would be different (iRBC-endothelial cells/RBC versus merozoite-RBC). Copy number variation (CNV) was also reported for *surf*_*4.1*_, but its biological significance is not known [2].

Population genetic approaches offer an avenue to study the effect of acquired immune responses on pathogen genetic polymorphism [6]. This strategy that involves study of nucleotide diversity for signatures of selection on particular antigens has unveiled candidate vaccine targets such as AMA1 [7–9], MSP1 [10], MSP2 [11, 12], MSP3 [13], EBA175 [14] and SURFIN_4.2_ [15] among others. *surf*_*4.1*_ gene region encoding SURFIN_4.1_ extracellular region possesses more polymorphic sites than the corresponding region of the *surf*_*4.2*_ gene. Significant positive deviations of Tajima’s *D* value from zero were detected on *surf*_*4.2*_ in both Thai and Kenyan parasite populations and positive selection was proposed on this gene [15, 16]. However, this is not the case for *surf*_*4.1*_, for which positively deviated Tajima’s *D* values were detected in Thai *P. falciparum* population [17], but negatively deviated values in the parasite population from Ngerenye area of Kilifi, eastern Kenya [15]. To gain insights on the cause of this difference, the authors determined *surf*_*4.1*_ gene sequences in the western Kenyan *P. falciparum* population and evaluated allelic diversity, the type of selection pressure, frameshift mutations, and copy number variation.

## Methods

### Parasite collection and DNA extraction

Twenty-three *P. falciparum* isolates obtained in the Lake Victoria islands of Kibuogi, Ngodhe, Takawiri and the shore village of Ungoye in Kenya in August 2014 [18] were adapted to the in vitro culture basically as described previously [19]; briefly, in RPMI-1640 medium containing 10% heat-inactivated pooled type AB^+^ human serum, 200 mM hypoxanthine, 20 µg/mL gentamicin and O^+^ human RBC at 2% hematocrit. Human erythrocytes and plasma used for the culture were obtained from Nagasaki Red Cross Blood Center. The sampling was authorized by the Ethical Review Committee of University of Nairobi and Kenyatta National Hospital and carried out in accordance with the approved guidelines. After a short period of culture (≤15 days, median 7 days), genomic DNA (gDNA) were extracted from the parasites using QIAamp DNA Mini Kit (Qiagen, Valencia, CA) according to the manufacturer’s instruction. Four isolates were cloned by limiting dilution before DNA extraction to determine the sequence encoding SURFIN_4.1_ (Gene ID PF3D7_0402200) extracellular region. To further analyse the frameshifts and CNV for the western Kenya parasites, DNA was obtained after cloning. For the cloned parasites, it took up to 40 days from starting culture to extracting DNA.

To evaluate the frameshifts and CNV of *surf*_*4.1*_ in Thai *P. falciparum* isolates, genomic DNA from 25 isolates and 3 clones prepared previously was used and showed clear single peaks for *surf*_*4.1*_ sequence [17]. The following Thai isolates were newly cloned before DNA extraction to avoid potential problems caused by the infection of more than one allele; MS807-H3, MS814-F1, MS815-A2, MS818-C2, MS819-A3, MS829-B5, MS833-B4, and MS844-A4.

### Polymerase chain reaction (PCR) amplification and sequencing

For 23 Kenyan samples (KK14-2, KK14-53, KK14-92, KN14-076, KN14-98, KT14-111, KT14-158, KU14-42, KU14-44, KU14-50, KU14-061, KU14-62, KU14-71, KU14-86, KU14-110, KU14-119, KU14-127, KU14-170, KU14-175, KU14-211, KU14-217, KU14-226, and KU14-257), DNA fragments for *surf*_*4.1*_ (PF3D7_0402200) encoding extracellular region were amplified with the following conditions; an initial denaturation step of 94 °C for 2 min followed by 40 amplification cycles of 98 °C for 10 s, 50 °C for 30 s, and 68 °C for 90 s, then final extension step of 68 °C for 5 min. Reaction was performed in a 12.5-μL reaction mixture containing 200 μM dNTP, 0.4 units of KOD-Plus NEO DNA polymerase (Toyobo, Japan), 2.0 μL of 10× KOD-Plus NEO buffer, 1.5 mM MgSO_4_, 0.4 μL of the template DNA solution, and 0.3 μM each of forward (F4.1, AAAGTTTTATTAAACCAGAAATGTAAAC, corresponding to the nucleotide positions (nt) −91 to −64) and reverse (R4.1, ATTACTTTGTTAAATAATATAAAAACG, nt 2375–2349) primers. PCR products were subjected to a 1.5% agarose gel electrophoresis and visualized with ethidium bromide under UV transillumination. Negative control reaction was always set using distilled water as a template solution. DNA standard marker was used to evaluate the size of the PCR products.

When the PCR band on the agarose gel was confirmed to be single with least or no background, amplified DNA fragments were treated with ExoSAP-IT (GE Healthcare, Buckinghamshire, UK) and sequenced directly with ABI PRISM^®^ BigDye™ Terminator ver1.1 using a panel of oligonucleotide primers as described before [17] according to the manufacturer’s instruction. Sequences were then analysed with an ABI3730 DNA analyzer (Applied Biosystems, Foster City, CA). The sample with multiple peaks in chromatogram, indicating a mixed allele infection, were sequenced after cloning of the PCR-amplified DNA fragment into pGEM^®^-T Easy (Promega, Madison WI) (KT14-111-T1, KU14-119-T1, KU14-119-T2) or sequencing was performed from the PCR products obtained from parasite lines after parasite cloning (KN14-076-B6, KT14-158-G3, KU14-061-B1, and KU14-257-A7). Sequences supported by at least two independent plasmid clones were employed. One isolate (KU14-119) yielded 2 distinct *surf*_*4.1*_ sequences (KU14-119-T1 and KU14-119-T2) that were supported by 2 and 4 independent plasmid clones, respectively.

To determine the existence of a frameshift at nt 2498–2503 in the *surf*_*4.1*_ gene encoding the region adjacent to the transmembrane region, PCR products were amplified with one of forward primers (F6, GATGGACTTAATGGATTTGATC, nt 1708–1729; F6_7G8, GATGAAAACGATGCATTTGTTC, nt 1708–1729; F6_HB3, TGATGAAAACAATGGATTTGTTC, nt 1707–1729; or F6_842, GTGGATAACTATGCATTTGTTC, nt 1708–1729) and a reverse primer (R1, TTATATCTTCTTCTTTCAAAACTTCATC, nt 2728–2701), and sequenced with R1 and R4.1. For some parasites, PCR products were amplified with F6 and R10 (CCAAACATATATCCAAAAATGTGCTC, nt 3520–3495), and sequenced with R10 and R11 (CCAACATTCTCCCTTGTCCATC, nt 2950–2929). To examine the other two frameshifts at nt 3894–3903 and 4529–4536 in the intracellular region, PCR products were amplified with primers F2 (GAAAAAGGATATTCCCATAAAACATTG, nt 2929–2950) and WR.R2 (CATTGCTATTTTTAACCAATCTG, nt 4891–4869) and sequenced with R2 and WR.R2.

### Quantitative real-time PCR (qPCR)

Copy number variation of *surf*_*4.1*_ gene was examined by the comparative *C*_T_ method using *ama1* gene as a control gene locus, for which CNV has not been noted. qPCR for *surf*_*4.1*_ was performed with the following conditions; an initial denaturation step of 95 °C for 10 min followed by 50 amplification cycles of 95 °C for 15 s, 45 °C for 20 s, and 62 °C for 1 min. Reactions were performed using Power SYBR Green Master Mix (Applied Biosystems) with primers PFD0100c.rtF2 (TAAGAACAGAACATAATTATGATAA, nt 308–332) and PFD0100.rtR1 (CAATCCTGTTCTGCATATTTTATG, nt 431–409) using Applied Biosystems 7500 Fast Real-Time PCR System. qPCR for *ama1* (PF3D7_1133400) was performed as same as for *surf*_*4.1*_ with a slight difference; with primers fAMA1-RT.F1 (AAGACGAAAATACATTACAACACGCA, nt 203–228) and fAMA1-RT.R1 (CTACTCTTATACCTGAACCATGAACT, nt 388–363) and the combined annealing/extension step at 62 °C for 1 min. The quality of the PCR-amplified products were validated by the melting curve method. The amplification efficiency of qPCR for both *surf*_*4.1*_ and *ama1* were determined to be 0.7 using serially diluted DNA solution of *P. falciparum* 3D7 line and used for the calculation.

### Statistical analyses

Sequences were aligned using a CLUSTAL W program [20]. The mean numbers of synonymous substitutions per synonymous site (*d*_S_) and nonsynonymous substitutions per nonsynonymous site (*d*_N_) and their standard errors were computed using the Nei and Gojobori method [21], with the Jukes and Cantor correction, implemented in MEGA4.0 [22]. The statistical difference between *d*_S_ and *d*_N_ was tested using a one-tailed Z-test with 500 bootstrap pseudo samples using MEGA. A value of *d*_N_ significantly higher than *d*_S_ at the 95% confidence level was taken as evidence for positive selection. When the number of synonymous (*Sd*) or nonsynonymous (*Nd*) differences were less than 10, Fisher’s exact test was used to estimate the significant difference. Nucleotide diversity (π) was computed using DnaSP5.0 [23]. Sliding window plots of the nucleotide diversity (90 bases with a step size of 3 bases) was computed using DnaSP5.0. Images of the sliding window plot results were generated using Excel software and modified using Adobe Photoshop. Nucleotide and amino acid (aa) positions are after 3D7 line sequence.

Tajima’s *D* test was used to evaluate a departure from the neutral evolution model by comparing θ (nucleotide diversity estimated based on the number of segregating site, S) and π (observed pairwise nucleotide diversity) to investigate whether polymorphic single nucleotide alleles tend to occur at higher or lower frequency than expectation under neutral drift [24]. Fu and Li’s D* and F* tests evaluate departures from neutrality by comparing the number of mutations in the external (considered to be “new” mutations) and internal (considered to be “older” mutations) branches of the genealogy. The number of external mutations would be deviated from neutral expectation by the selective pressure, whereas the number of internal mutations is less affected. Under positive balancing selection, the number of internal “old” mutations is expected to be higher than the number of external “new” mutations. Fu and Li’s D* compares the estimated θ based on the number of singletons (mutations appearing only once among the sequences, which is new and locates in the external branches) and that based on S. Fu and Li’s F* compares the estimated θ based on the number of singletons and that based on k (average number of pairwise nucleotide difference) [25]. These tests were executed using DnaSP5.0.

## Results

### Polymorphism of the *surf*_*4.1*_ gene and its product in the western Kenyan *P. falciparum* population

**Fig. 2:**
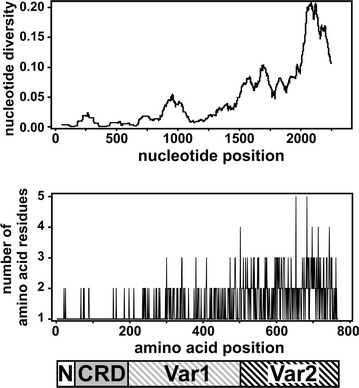
Sliding window plot of nucleotide diversity and amino acid polymorphism of SURFIN_4.1_ extracellular region in the western Kenyan *Plasmodium falciparum* population. Window length of 90 bp and step size of 3 bp is used for the sliding window plot (*top*). The number of the amino acid type at each amino acid position (*middle*) and a scheme of SURFIN_4.1_ extracellular region (*bottom*) are shown in scale to visualize the location of the polymorphic sites. SURFIN_4.1_ extracellular region was divided into 4 parts: N-terminal (N), Cys-rich domain (CRD), and variable regions 1 and 2 (Var1 and Var2). A total of 24 sequences from western Kenya isolates were used. Nucleotide and amino acid positions are after 3D7 line sequences

Twenty-four nucleotide sequences (nt 4–2289) encoding the extracellular region of SURFIN_4.1_ were obtained from western Kenyan field isolates in this study. A total of 410 polymorphic nucleotide sites were observed with an average pairwise nucleotide diversity of 0.048. All insertions/deletions (indels; AAT at nt 1348–1350, TAC at 1746–1748, GGA at 2272–2274) found in previously reported Thai *surf*_*4.1*_ sequences were also detected in western Kenyan isolates. To evaluate the regions accumulating the polymorphisms, the extracellular region of SURFIN_4.1_ was divided into 4 regions based on amino acid sequence conservation among SURFIN members: N-terminal segment (Nter; aa 1–50, nt 1–150), Cys-rich domain (CRD; aa 51–195, nt 151–585), a variable region 1 (Var1; aa 196–502, nt 586–1506), and −2 (Var2; aa 503–765, nt 1507–2289) as described previously [17]. The polymorphic sites were mainly distributed in the variable region; 398 among 410 polymorphic nucleotide sites were located in the variable region and 11.7% of 918 nucleotides consisting Var1 region and 37.2% of 783 nucleotides consisting of Var2 region were polymorphic (Table 2). The degree of the polymorphism was enhanced at amino acid level; 26.0% of 307 amino acids consisting of Var1 and 66.6% of 261 amino acids consisting of Var2 were polymorphic. Sliding window plots of nucleotide diversity and amino acid polymorphism indicated that in the Var2 region more polymorphism accumulated toward the C-terminal side as similar as the previous finding for Thai parasite population (Fig. 2) [17].

### Selection on the *surf*_*4.1*_ gene in the western Kenyan *P. falciparum* population

**Fig. 3:**
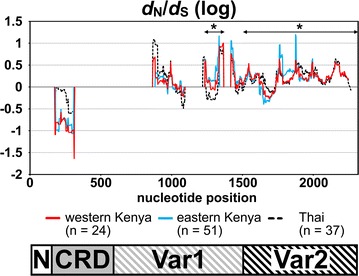
Sliding window plots of *d*_N_/*d*_S_ ratio for *Plasmodium falciparum surf*_*4.1*_ sequence encoding extracellular region. Data obtained in this study for western Kenyan isolates (n = 24) is plotted with a *solid red line*. As a comparison, previously published *surf*_*4.1*_ sequences from eastern Kenya [15] and Thai [17] are plotted with a *solid cyan line* and a *dotted black line*, respectively. *Asterisks* indicate a part of Var1 region and Var2 region where *d*_N_ was significantly higher than *d*_S_ in two Kenyan parasite populations (p < 0.05). Nucleotide positions are after 3D7 line sequence. Window length is 90 bp and step size is 3 bp. A scheme of SURFIN_4.1_ extracellular region (*bottom*) are shown to visualize the location of the peaks. N-terminal (N), Cys-rich domain (CRD), and variable regions 1 and 2 (Var1 and Var2)

The signatures of selection on *surf*_*4.1*_ were first evaluated in the western Kenyan parasite population by comparing *d*_S_ and *d*_N_ (Table 1). Significant excess of *d*_N_ over *d*_S_ was observed when the entire sequence or only Var2 region were analysed (p = 0.004 and 0.0003, respectively). No significant difference between *d*_N_ and *d*_S_ was found in Nter, CRD, and Var1 regions (Fisher’s exact test). To visualize the sites where the excess of *d*_N_ over *d*_S_ is observed and compare the pattern among 3 different geographical locations, the sliding window plots of *d*_N_/*d*_S_ calculated for the *surf*_*4.1*_ gene sequences previously reported from the eastern Kenyan (n = 51) and Thai (n = 37) *P. falciparum* populations were overlaid in Fig. 3 [15, 17]. All three populations showed a similar pattern and two Kenyan populations showed a significant excess of *d*_N_ over *d*_S_ at the Var1 C-terminus and almost entire Var2 region (p < 0.05).

**Table 1:** Nucleotide diversity of *Plasmodium falciparum surf*_4.1_ in western Kenyan isolates (n = 24)

Region (position)	Number of sites (base)	Indels	k	*Nd*	*N*	*Sd*	*S*	π	*d*_N_	*d*_S_	*d*_N_/*d*_S_	*p*
			(SE)	(SE)	(SE)	(SE)	(SE)	(SE)	(SE)	(SE)		(*d*_N_ > *d*_S_)
Extracellular	2286	9	104.64	87.22	1791.60	17.43	488.40	0.048	0.051	0.037	1.38	0.004
(4–2289)			(5.50)	(6.03)	(8.40)	(2.25)	(8.40)	(0.003)	(0.003)	(0.005)		
Nter	147	0	0.24	0.24	118.30	0.00	28.70	0.002	0.002	0.000	∞	ns*
(4–150)			(0.19)	(0.17)	(2.01)	(0.00)	(2.10)	(0.001)	(0.002)	(0.000)		
CRD	435	0	2.99	1.52	351.61	1.47	80.39	0.007	0.004	0.019	0.21	ns*
(151–585)			(1.11)	(0.70)	(3.68)	(0.79)	(3.64)	(0.003)	(0.002)	(0.011)		
Var1	918	3	20.23	17.86	721.42	2.37	196.58	0.023	0.026	0.012	2.17	ns*
(586–1506)			(2.15)	(2.16)	(5.52)	(0.64)	(5.57)	(0.002)	(0.003)	(0.003)		
Var2	783	6	81.19	67.60	600.28	13.59	182.72	0.115	0.126	0.080	1.58	0.0003
(1507–2289)			(4.73)	(4.62)	(4.74)	(1.97)	(4.57)	(0.007)	(0.009)	(0.011)		

Extracellular, extracellular region; Nter, N-terminal segment; CRD, cysteine-rich domain; Var1, variable region 1; Var2, variable region 2; sites, sites nucleotide analyzed; indels, insertion/deletion polymorphism; k, the average number of nucleotide differences; *N* and *S*, average numbers of nonsynonymous and synonymous sites; π, pairwise nucleotide diversity (Jukes-Cantor model); *d*_N_, mean number of nonsynonymous substitutions per nonsynonymous site; *d*_S_, mean number of synonymous substitutions per synonymous site; SE, standard error computed using the Nei-Gojobori method with the Jukes-Cantor correction. SE was estimated using the bootstrap method with 500 replication

The numbers of synonymous (*Sd*) and nonsynonymous (*Nd*) differences were calculated by the Nei-Gojobori method. *p* value indicates the statistical difference between *d*_N_ and *d*_S_, tested using one-tail Z-test with 500 bootstrap pseudo samples implemented in MEGA. ns indicates not significant by two-tailed Fisher’s exact tests (*). Number is after 3D7 line sequence

**Fig. 4:**
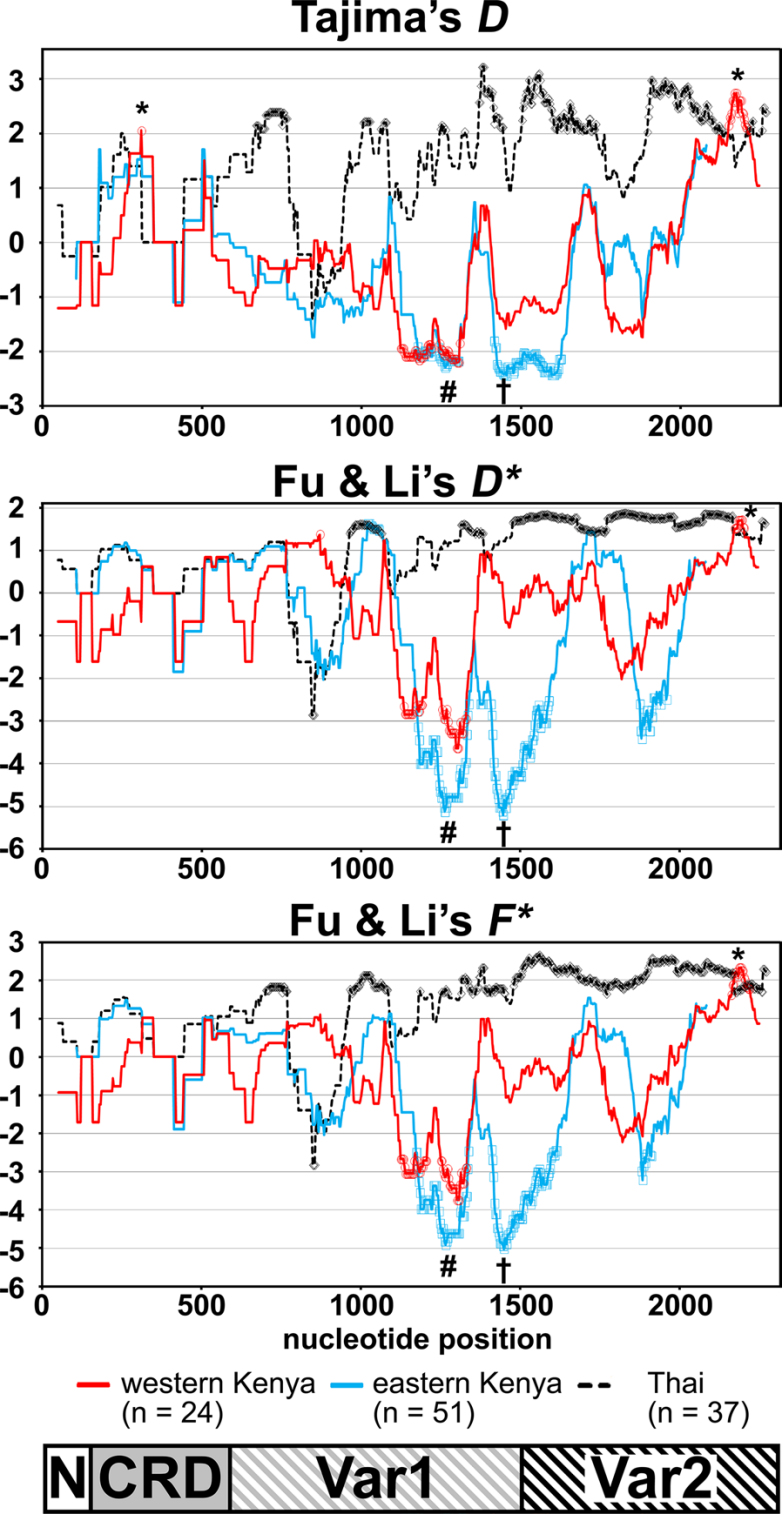
Sliding window plots of Tajima’s *D*, Fu and Li’s D* and F* for *Plasmodium falciparum surf*_*4.1*_ sequence encoding the extracellular region. Data obtained in this study for western Kenyan isolates (n = 24) is plotted with a *solid red line*. As a comparison, previously published *surf*_*4.1*_ sequences from eastern Kenya [15] and Thai [17] are plotted with a* solid cyan line* and a *dotted black line*, respectively. Sites significantly departed from the neutrality (p < 0.05, two-tailed) are indicated with *circle*, *square*, and *diamond* symbols on each line. *Asterisk* and *hash symbols* indicate the region where positive or negative deviation from the neutrality, respectively, were detected in the western Kenyan parasite population in this study. *Dagger symbol* indicates the region where negative deviation from the neutrality was detected in the eastern, but not western Kenyan population [15]. Nucleotide positions are after 3D7 line sequence. Window length is 90 bp and step size is 3 bp. A scheme of SURFIN_4.1_ extracellular region (*bottom*) are shown to visualize the location of the peaks. N-terminal (N), Cys-rich domain (CRD), and variable regions 1 and 2 (Var1 and Var2)

No significant deviation from the neutrality was detected by 3 population-based tests when the entire obtained sequence was analysed, or the 4 subdivided regions were analysed separately (Table 2). To analyse further the region where the potential balancing/directional selection act on the *surf*_*4.1*_ gene and compare among 3 different geographical locations, the sliding window plots of Tajima’s *D*, Fu and Li’s D*, and F* calculated for *surf*_*4.1*_ gene sequences previously reported from the eastern Kenyan and Thai *P. falciparum* populations were overlaid in Fig. 4. All 3 population-based tests showed similar patterns between western and eastern Kenyan populations and revealed a significant positive deviation of greater than zero at the Var2 C-terminus (p < 0.05) in the western Kenyan population, where Thai population also showed significant positive Fu and Li’s D*, and F* values and not significant but positive Tajima’s *D* value (0.05 ≤ p < 0.1) [17]. This region was not sequenced for the eastern Kenyan population, thereby not examined in the previous report [15]. Thus, both Kenyan and Thai populations consistently showed positive deviation on the Var2 C-terminus. Tajima’s test also detected significant positive deviation on CRD region (p < 0.05, Fig. 4 asterisk) in the western Kenyan population, where not significant but positive values are similarly seen in both eastern Kenyan and Thai populations (p ≥ 0.1). In addition, significant negative deviation was detected on the C-terminal half of the Var1 region (p < 0.05, Fig. 4 hashes), where significant negative deviation was also detected in the eastern Kenyan population [15], but positive selection was detected in the Thai population. Significant negative deviation observed in the region encoding the very C-terminal side of Var1 to N-terminal of Var2 region in the eastern Kenyan population (Fig. 4 dagger) was not seen in the western Kenyan population.

**Table 2:** Test of neutrality for *Plasmodium falciparum surf*_4.1_ for the western Kenyan isolates (n = 24)

Region	Nucleotide position	Number of sites (base)	η	S	Two variants		More than two variants	π	θ	Tajima’s *D*	Fu and Li’s	
					Singleton	Not singleton					D*	F*
Extracellular	4–2289	2286	439	410	115	266	29	0.046	0.048	−0.19	−0.29	−0.30
Nter	4–150	147	2	2	1	1	0	0.002	0.004	−1.20	−0.66	−0.93
CRD	151–585	435	11	10	3	6	1	0.007	0.006	0.39	−0.61	−0.36
Var1	586–1506	918	109	107	44	61	2	0.022	0.031	−1.17	−0.93	−1.18
Var2	1507–2289	783	317	291	67	198	26	0.104	0.100	0.17	−0.02	0.05

Sequence number is after 3D7 line sequence

Extracellular, extracellular region; Nter, N-terminal segment; CRD, cysteine-rich domain; Var1, variable region 1; Var2, variable region 2; sites, nucleotide sites analyzed; η, the total number of mutations; S, number of segregating sites; π, observed nucleotide diversity; θ, the expected nucleotide diversity under neutrality derived from S

**Fig. 5:**
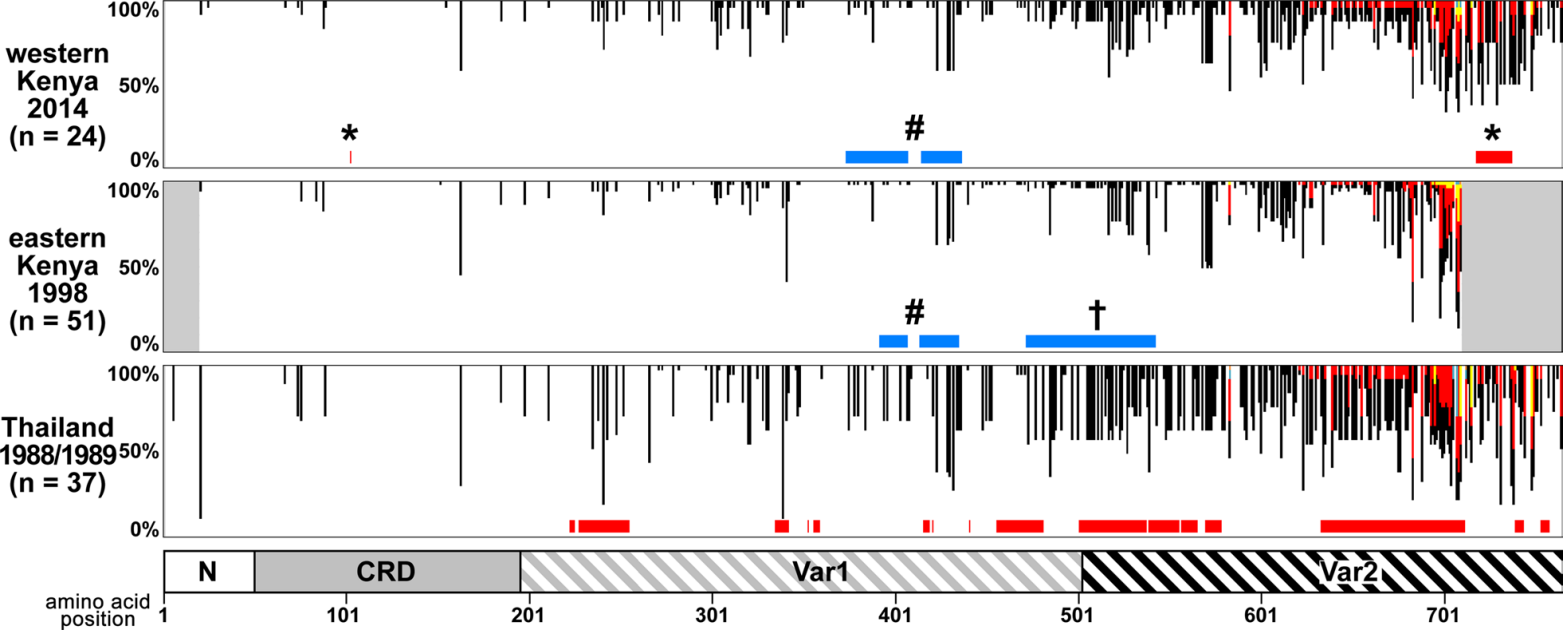
Frequency distribution of amino acid of *Plasmodium falciparum* SURFIN_4.1_ extracellular region. Data obtained in this study for western Kenya isolates in 2014 (n = 24), eastern Kenya isolates in 1998 (n = 51) [15], and Thai isolates in 1988/1989 (n = 37) [17] are plotted. Each allele at polymorphic sites are shown with *white*, *black*, *red*, *yellow*, and *blue*. Positions where Tajima’s *D* showed significant positive or negative deviation from the neutrality (p < 0.05) are indicated with *red* or *blue bars*, respectively. *Asterisk symbols* indicate the region where positive deviation from the neutrality was detected in the western Kenyan parasite population in this study. *Hash* and *dagger symbol* indicates the region where negative deviation from the neutrality was detected. A scheme of SURFIN_4.1_ extracellular region (*bottom*) are shown to visualize the location of the polymorphic sites. N-terminal (N), Cys-rich domain (CRD), and variable regions 1 and 2 (Var1 and Var2)

To understand the difference in the selection detected on the *surf*_*4.1*_ gene locus between Kenyan and Thai *P. falciparum* populations, the pattern and frequency distribution of the polymorphic amino acid sites were visualized in these populations (Fig. 5). Both Kenyan populations (eastern Kenya, 1998, reported previously [15] and western Kenya, 2014, obtained in this study) had similar distribution of polymorphic amino acid sites suggesting that the pattern is spatially and temporally stable between these two areas in Kenya. When the frequency of the minor allele at 181 dimorphic amino acid sites were compared, it is evident that Kenyan populations possess less dimorphic amino acid sites at intermediate frequency whereas the Thai population (1988–1989, reported previously [17]) were skewed towards more frequent intermediate alleles.

### Frequency of the frameshift mutation that would result no SURFIN_4.1_ expression on the iRBC surface varies between western Kenyan and Thai parasite populations

Frameshift mutations influence the expression of SURFIN_4.1_ on the surface of iRBC, potentially impacting evolutionary dynamics. To infer on this possibility, the frequency of the frameshift mutations was examined at nt 2498–2503, 3894–3903, and 4529–4536 in *P. falciparum* lines isolated from western Kenya and Thailand (Fig. 1; Table 3). No parasite line showed a frameshift at nt 4529–4536. Twenty-two among 23 western Kenyan parasite lines (96%) and 22 among 36 Thai parasite lines (61%) possessed a frameshift at nt 2498–2503 that would produce SURFIN_4.1_ without cytoplasmic WR domains. No western Kenyan, but one Thai parasite line possessed a frameshift at nt 3894–3903 that would produce SURFIN_4.1_ with single WR domain. Hence, one western Kenyan and 13 Thai lines did not possess frameshifts at the examined 3 sites as observed in IT line sequence and possibly express SURFIN_4.1_ with 3 intact WR domains. Therefore, only 1 among 23 western Kenyan parasites (4%) and 14 among 36 Thai parasites (39%), are expected to possess SURFIN_4.1_ with at least one cytoplasmic WR domain.

**Table 3:** Existence of frameshift mutations in the *surf*_*4.1*_ region encoding the cytoplasmic region in the western Kenyan and Thai *Plasmodium falciparum* isolates

	Frameshift (nucleotide positions^a^)			Number (%) of parasite line/clone^b^			
	2498–2503	3894–3903	4529–4536	Western Kenya		Thai	
Pattern 1	Yes	Yes	No	8	(35)	17	(47)
Pattern 2	Yes	No	No	14	(61)	5	(14)
Pattern 3	No	Yes	No	0	(0)	1	(3)
Pattern 4	No	No	No	1	(4)	13	(36)
Total				23	(100)	36	(100)
3D7^c^	Yes	Yes	No				
FCR3^c^	No	No	Yes				
IT^d^	No	No	No				
PrCDC^d^	No	No	No				

^a^ Nucleotide positions are after 3D7 line sequence

^b^ Pattern 1 contains KK14-92-B7, KN14-098-D1, KT14-111-H2, KU14-044-D2, KU14-050-B3, KU14-071-D4, KU14-226-G5, KU14-257-A7, MS804, MS807-H3, MS836, MS808, MS812, MS828, MS827, MS838, MS830, MS814-F1, MS814-H1, MS815-A2, MS818-C2, MS833-B4, MS840, MS841, and MS829-B5; pattern 2 contains KK14-02-B9, KK14-53-F6, KN14-076-B6, KT14-158-G3, KU14-042-D12, KU14-061-B1, KU14-062-A8, KU14-110-C4, KU14-119-D4, KU14-127-B12, KU14-170-A10, KU14-175-D6, KU14-211-D1, KU14-217-B5, MS811, MS813, MS835A1, MS844-A4, and MS947; pattern 3 contains MS843; and pattern 4 contains KU14-086-B3, MS805, MS806, MS809, MS810, MS816, MS817, MS819-A3, MS822-G8, MS825, MS837, MS842, MS946, and MS948

^c^ GenBank accession numbers for 3D7 and FCR3 lines are AL844503 and AB759920, respectively

^d^ PFIT_0400900 and PRCDC_0005300 from GeneDB, Wellcome Trust Sanger Institute, UK (both are 2015-06-18 version)

To gain insight if these frameshifts are common in the giant ape malaria parasites close to *P. falciparum* [26], *surf*_*4.1*_ gene sequence (PRCDC_0005300, GeneDB) of the CDC line of Chimpanzee malaria parasite *Plasmodium reichenowi* was examined. It was found that *P. reichenowi surf*_*4.1*_ sequence did not possess these frameshifts even though only one sequence was examined, suggesting that at least an intact *surf*_*4.1*_ gene without frameshifts existed in the common ancestor of *P. falciparum* and *P. reichenowi* and likely not rare.

### *surf*_*4.1*_ CNV is common in the western Kenyan *P. falciparum* population, but not in Thai population

Recent genome-wide analysis of the *P. falciparum* CNVs suggested that CNVs were targets of the purifying selection and particular CNVs found at high frequencies in populations with a large effective population size (such as Africa) were likely beneficial [27]. Because CNV has been reported for *surf*_*4.1*_, the copy number was examined in western Kenyan and Thai isolates. More than one copy of *surf*_*4.1*_ gene was detected in western Kenya lines (17%, 3/24 showed two copies and 1/24 showed thee copies), but not in Thai lines (0%, 0/36). This suggests that even though the cytoplasmic WR domain was not expressed, *surf*_*4.1*_ CNV appears to be positively selected in Africa. As a control, 3D7 and FCR3 lines were examined and single and three copies were detected, respectively. Although Mphande et al. detected 6 copies of *surf*_*4.1*_ in FCR3, CNVs is known to rapidly change during the in vitro culture, so this may be due to the different culture history of FCR3 [3].

## Discussion

In this study, *P. falciparum* field isolates were collected from patients in western Kenya, adapted to in vitro culture, and 24 sequences encoding SURFIN_4.1_ extracellular region were determined. Despite the previous report describing only negative selection on *surf*_*4.1*_ in the eastern Kenyan parasite population [15], positive balancing selection was detected on this gene in the western Kenyan population. The highest positive deviation by all neutrality tests were detected on the stretch encoding a region close to the transmembrane region (nt 2108–2254 by Tajima’s test, nt 2129–2248 by Fu and Li’s D* test, and nt 2117–2263 by Fu and Li’s F* test). Because Ochola et al. [15] analysed *surf*_*4.1*_ sequences spanning only nt 61–2124, the most polymorphic region was not fully covered by their analysis, explaining the failure to detect positive selection. It was infered that the Var2 C-terminus is under immunological selection pressure in both Kenyan and Thai populations.

Negative values were detected on the *surf*_*4.1*_ gene encoding Var1 C-terminus by population-based analyses in the western Kenyan parasite population, which were also detected for eastern Kenyan population and inferred to be a reflection of directional selection [15]. However, positive values were detected for this region in Thai population [17]. To seek possible explanation for this discrepancy, the study examined frameshifts in the cytoplasmic region that cause a *surf*_*4.1*_ product having only 19 aa cytoplasmic tail without WR domain, which is implicated to be responsible for the iRBC surface exposure. Ninety-six percent of Kenyan *P. falciparum* lines possessed this frameshift mutation and were expected to be exposed to the host immunity only on the merozoite surface. If SURFIN_4.1_ exposed on the iRBCs is involved with the adherence to the endothelial cells or uninfected RBCs, the frequency of the observed alleles is a result of a balance between pressures to increase the alleles with most fitted specificity and strength to recognize the receptor and the other pressure to escape from the host immunity. Because Var1 region may participate to form a homomeric complex [4], dominant alleles in Kenya may have a better fitness to function on the merozoite surface.

**Fig. 6:**
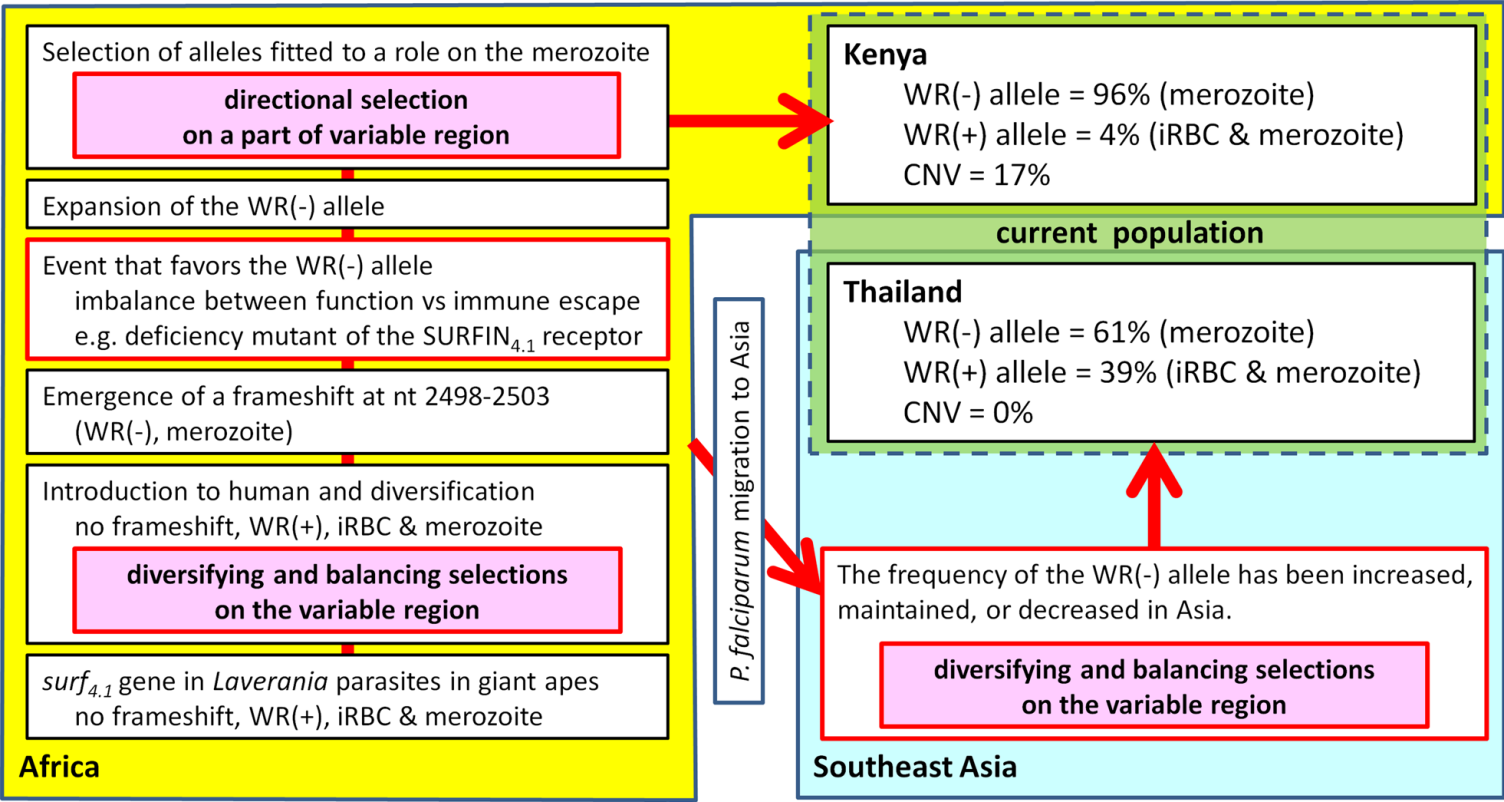
Proposed model for the evolution of the *surf*_*4.1*_ gene locus. Nucleotide (nt) positions are after 3D7 line sequence. *CNV* copy number variation, *iRBC* infected red blood cell, *WR* Trp-rich domain

Based on the finding in this study, the authors propose the following model for the *surf*_*4.1*_ gene evolution in Kenya and Thailand (Fig. 6). Ancestor of *surf*_*4.1*_ gene with intact full length of open reading frame was originally formed in *Laverania Plasmodium* infecting giant apes in Africa [26], as suggested by the sequence of the *P. reichenowi surf*_*4.1*_ ortholog without frameshift mutations. After the introduction of the ancestral *P. falciparum* to human population, most of the currently observed *surf*_*4.1*_ polymorphism was shaped under the diversifying selection in Africa, which is suggested by the significant excess of *d*_N_ over *d*_S_ and the conserved polymorphism between Kenyan and Thai parasite isolates. It should be noted that the polymorphism of SURFIN_4.1_ is more abundant than that of SURFIN_4.2_ that is still expressed on the iRBC, suggesting stronger immune pressure to SURFIN_4.1_ [17]. Frameshift mutation that results in truncated SURFIN_4.1_ expressed on the merozoite surface but not on the iRBC emerged and expanded in Africa. Although the cause of increased frequency of this frameshift mutation in the *P. falciparum* population is unclear, a loss-of-function of SURFIN_4.1_ on the iRBC surface (such as a deficient mutant of the host receptor) followed by immunity against non/less-functional SURFIN_4.1_ would favour this frameshift mutation. Such a hypothesis is plausible as it is known that some human molecules were evolved to escape from the parasite recognition, for example majority of west Africans do not express the Duffy Antigen Receptor for Chemokines (DARC), a receptor for the other human malaria parasite *Plasmodium vivax*, on their RBC and became resistant against this malaria parasite species [28, 29]. Acquiring a role only on the merozoite surface, fitted SURFIN_4.1_ alleles might have been selected in Africa under directional selection. CNVs would be beneficial under such situation. In Thailand, it is not clear if the frequency of this frameshift mutation was increased, maintained, or decreased to ~60% after the *P. falciparum* migration to Asia, and also if the frequency is still increasing toward the level in Africa (~95%). The transmission intensity in Southeast Asia is much lower than Africa, reducing the chance to mate and produce novel haplotypes, which may influence the speed to change the frequency of particular alleles that fit to the new environment. However, because *P. falciparum* was speculated to migrate to Asia with humans around ~50,000–60,000 years ago [30], the intermediate frequency of this frameshift mutation observed in Thailand may be already adapted to the local environment. If this is the case, the hypothetical SURFIN_4.1_ receptor may be still expressed in the Asian human population. Nonetheless, more studies are needed to understand the background of this complex genetic signature, especially identification of the hypothetical SURFIN_4.1_ receptor would provide a critical answer to this question.

## Conclusions

Signatures of diversifying and balancing selections were detected on the *surf*_*4.1*_ region encoding SURFIN_4.1_ Var2 C-terminus in western Kenyan *P. falciparum* population similar to Thai parasite population [17]. Whereas, signature of directional selection was detected on the region encoding SURFIN_4.1_ Var1 C-terminus in the western Kenyan population, consistent to the previous report for the eastern Kenyan population [15]. Most western Kenyan isolates possess a frameshift mutation that would limit the expression of SURFIN_4.1_ on the merozoite, but only ~60% of Thai isolates possess this frameshift, which would significantly affect the level and type of the selection pressure against this protein. CNV was observed in 4/24 Kenya isolates and 0/36 Thai isolates, which may be a consequence of this frameshift mutation and to increase expression on the merozoite surface.

## Abbreviations

aa: amino acid position
CNV: copy number variation
DNA: deoxyribo nucleic acid
indels: insertions/deletions
nt: nucleotide position
PCR: polymerase chain reaction
surf gene: surface-associated interspersed gene
